# Case-Cohort Conundrum: Fish or No Fish?

**Published:** 2015-07-01

**Authors:** Kelley D. Mayden

**Affiliations:** Southwest Virginia Cancer Center, Norton, Virginia, and King University, Bristol, Tennessee

Oncology advanced practitioners (APs) are advocates for and witnesses to the increasing interest in self-regulation of health care by patients. Data from the 2007 National Health Interview Survey (a cross-sectional household interview) suggest that one way in which patients with cancer attempt to manage their health is through the use of complementary and alternative medicine (CAM).

The use of CAM is estimated to be as high as 64% in the cancer population, and predictors of CAM use include female gender, stage of disease, age, education, income, race, and geographic location (Anderson & Taylor, 2012). Statistics from the survey document the frequent use of herbal supplements among respondents with and without cancer. For example, 44% of respondents with cancer and 38% of respondents without cancer reported the use of fish oils in the past 12 months prior to survey completion (Barnes, Bloom, & Nahin, 2008).

Initially, such statistics on the use of fish oils may have been of more interest to cardiovascular providers, but the use of fish oils in the oncology setting is becoming a topic of interest and debate. This is particularly true when it comes to understanding the relationship between long-chain omega-3 fatty acids (eicosapentaenoic acid [EPA], docosapentaenoic acid [DPA], and docosahexaenoic acid [DHA]) and prostate cancer risk. Data are conflicting, and a complete body of evidence for or against the use of omega-3 supplementation is lacking.

Advanced practitioners are increasingly being asked by patients to commit to a recommendation for or against fish oil supplementation, especially among men with prostate cancer. Much of this attention follows the media sensationalism of the published finding by Brasky et al. suggesting an increased risk of prostate cancer among men with high blood concentrations of long-chain omega-3 fatty acids (Brasky et al., 2013). These findings have prompted many patients to alter dietary intake of fish and abandon the use of fish oil supplements. The estimated 220,800 new cases of prostate cancer and 27,540 cases of prostate cancer death in 2015 (American Cancer Society) mandate the need for APs to examine the evidence surrounding omega-3 usage in relation to prostate cancer prevention and risk prior to providing guidance to patients about omega-3 dietary intake or supplement usage.

## EVALUATING THE CLINICAL EVIDENCE: NOT ALL STUDIES ARE CREATED EQUAL

The danger of a single study directing clinical or patient practice is that it is not representative of the combined body of evidence. Each study must be examined in terms of validity and dependability. This fact is echoed in the article by Marilyn Haas-Haseman beginning on page 376 which covered the findings by Brasky et al. In this companion article, I will examine the strengths and weaknesses of the Brasky et al. study, suggest directions for similar trials in the future, and provide some reference for evidence to guide APs in recommendations for or against the use of omega-3 fatty acids among patients at risk for or with prostate cancer.

Compared to conventional medicine, evidence from randomized controlled trials (RCTs) in CAM is lacking. This is due in part to a discrepancy in funding and characteristics that make the design of RCTs in CAM difficult (see Table 1).

**Table 1:**
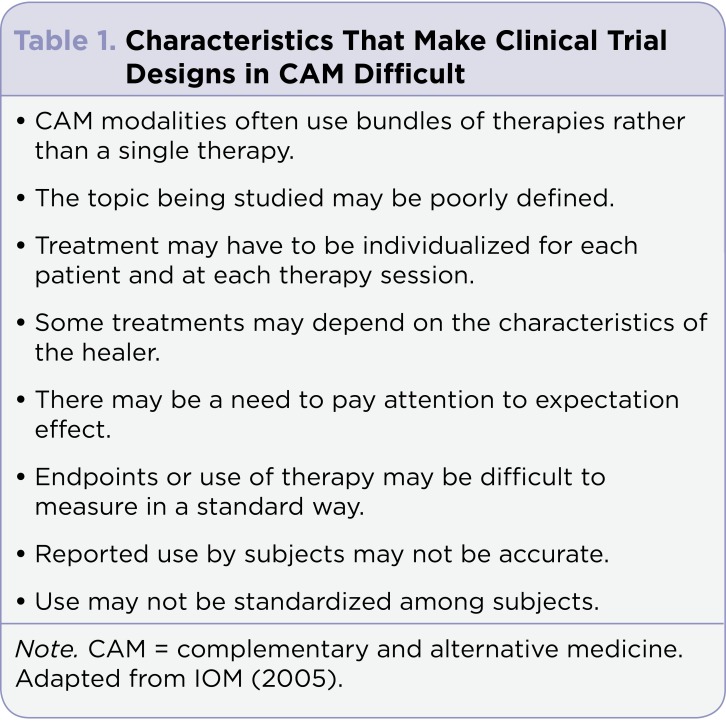
Characteristics That Make Clinical Trial Designs in CAM Difficult

Extrapolation of data from clinical trials not specifically designed to randomly explore complementary interventions can weaken the CAM body of evidence and does little to add to the accumulation of evidence that will form the backbone of decision-making. Case in point, in their study, Brasky and colleagues suggest caution when advocating an increase in the use of long-chain fatty acids based on information obtained from the SELECT trial. This trial was an RCT designed to examine the relationship between selenium and vitamin E, individually or together, and prostate cancer incidence, and was not initially designed to evaluate a cause-and-effect relationship of long-chain fatty acids to prostate cancer incidence (Dunn, Richmond, Minasian, Ryan, & Ford, 2010). This type of data extrapolation invites room for criticism.

Randomized controlled trials are the gold standard for evaluating the safety, efficacy, and tolerability of new medical therapies or interventions. Direct determination of cause and effect is best proven with this type of trial. Randomized trials may be classified by study design (see Table 2).

**Table 2:**
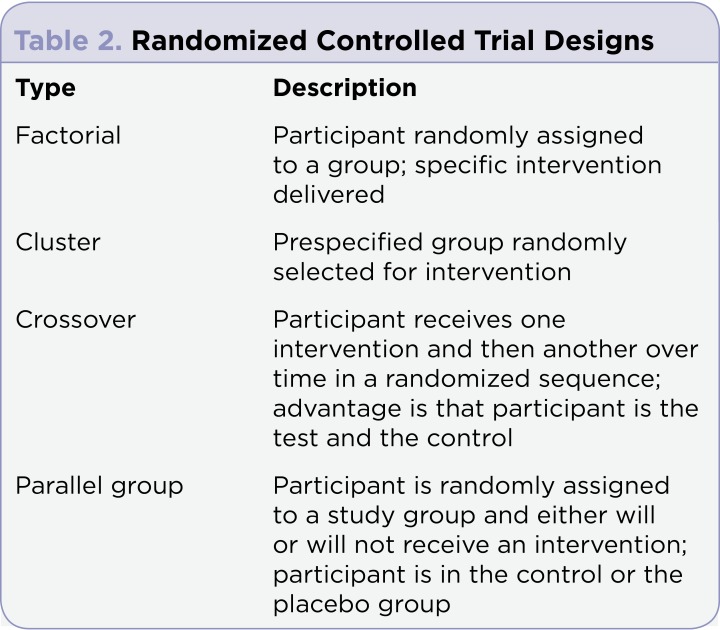
Randomized Controlled Trial Designs

A key element in RCTs includes randomization between a group of participants intended to receive intervention or testing and a control or placebo group not intended to receive intervention. Randomization occurs once eligibility has been determined and is followed by the intervention being studied. Eligibility criteria are outlined in an approved study protocol. The control and comparison groups are then followed for a prespecified period. Predetermined endpoints are measured based on data analysis, conclusions are formulated, and the findings are then presented orally or appear in the literature.

The CONSORT Statement is an evidence-based set of recommendations to guide reporting of RCT findings and contains key elements, as outlined in Table 3. Randomization can minimize confounding factors and decrease biases. Disadvantages to RCTs include expense and limited application to real-world situations due to study population characteristics or measured outcomes (Besen & Gan, 2014).

**Table 3:**
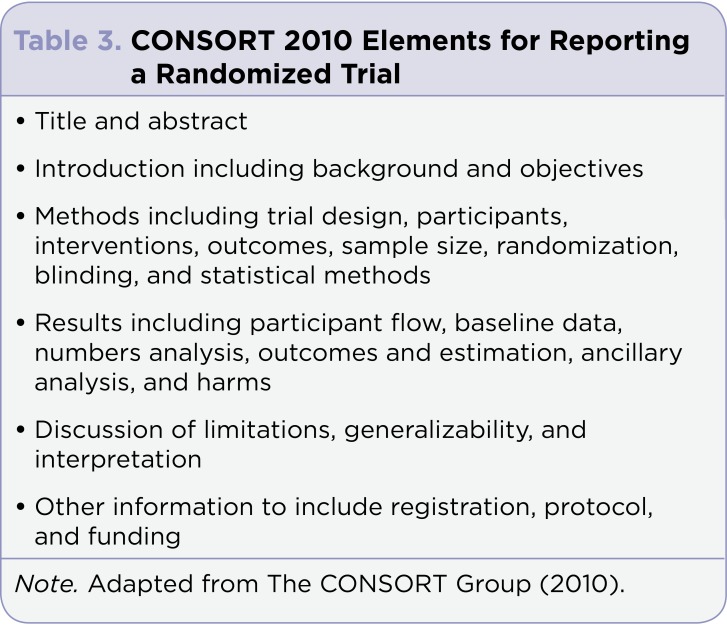
CONSORT 2010 Elements for Reporting a Randomized Trial

The Brasky study was a case-cohort design without direct intervention. Cohort studies can provide valid correlations between cause and effect. A cohort is a select group of individuals who share common characteristics or exposures without the disease or outcome of interest who will be followed over time to determine the incidence of the disease or outcome of interest (Besen & Gan, 2014). Brasky and colleagues chose a case cohort of 1,393 men from the SELECT trial; 834 of them were diagnosed with prostate cancer.

Case-cohort studies can be prospective, from present to future, or retrospective, looking from the present to the past (historical). Prospective studies are designed with specific data-collection methods and are typically more complete, but one disadvantage is the long follow-up period while observing for events or disease occurrences (Song & Chung, 2010). On the other hand, retrospective studies are less expensive and provide an immediate data bank. Analysis of the data provides an opportunity to observe relationships between exposure and incidence. Disadvantages of retrospective studies include limited control over data collection and an inability to identify true causality (Besen & Gan, 2014).

The mixture of observations from the SELECT trial and a meta-analysis from previous prospective biomarker studies of fatty acids and prostate cancer risk does not support direct causality and is structurally weak in terms of supporting the bold association of omega-3 fatty acids to prostate cancer. It does little to incorporate evidence from other studies, which did not find an increased risk for prostate cancer from use of omega-3 fatty acids. In fact, in their earlier study, Brasky et al. did not correlate the risk of prostate cancer with EPA, but only with DHA (Brasky et al., 2011).

**Comparison With Other Studies**

Studies from Aronson et al. (2011), Augustsson et al. (2003), Bosire et al. (2013), Chavarro et al. (2007), and Norrish et al. (1999) support the use of omega-3 fatty acids for prostate cancer risk reduction. In their review and meta-analysis, Szymanski, Wheeler, and Mucci (2010) found no strong evidence of a protective association of fish consumption with prostate cancer incidence but did find that fish consumption was associated with a 63% reduction in prostate cancer–specific mortality.

Leitzmann et al. (2004) reported an increase in the risk of advanced prostate cancer from the use of alpha-linolenic acid but a reduction in the risk of total and advanced prostate cancer with the intake of EPA and DHA. Mannisto et al. (2003) failed to demonstrate an association between EPA or DHA and prostate cancer risk. Park et al. (2009) reported a nonsignificant trend toward an increased risk of advanced prostate cancer with EPA only, whereas Harvei et al. (1997) reported a nonsignificant trend toward reduced risk of prostate cancer with increased levels of plasma phospholipid DPA. Crowe et al. (2008) measured a positive association between EPA and high-grade prostate cancer but not low-grade, localized, or advanced prostate cancer.

A Swedish study that examined dietary fatty acid intake among 525 men and prostate cancer survival suggested that high marine omega-3 fatty acid intake may improve disease-specific survival for all men (Epstein et al., 2012). A recent dose-response meta-analysis of prospective observational studies examining both dietary intake and circulating omega-3 polyunsaturated fatty acids on prostate cancer risk proposed a marginal positive association of risk with blood concentrations of DHA but not EPA.

Subgroup analyses showed that blood EPA concentration was positively associated with aggressive prostate cancer risk, whereas blood DHA concentration correlated with nonaggressive prostate cancer risk (Fu, Zheng, Yang, & Li, 2015). Interestingly, Japanese men consume much higher levels of omega-3 fatty acids, yet they have a lower rate of age-adjusted mortality from prostate cancer compared with men in the United States (Wynder, Fujita, Harris, Hirayama, & Hiyama, 1991).

These studies represent an abridged summary of the current available evidence. They demonstrate that the current body of evidence does not support a clear-cut recommendation for or against the use of omega-3 fatty acids in men with or at risk for prostate cancer.

In hindsight, conclusions from the Brasky study would have better supported a recommendation for or against use of omega-3 fatty acid in relation to prostate cancer risk if there were some measure of direct intervention with long-chain fatty acids among patients with prostate cancer and a control group. Future trials with a randomized design evaluating omega-3 fatty acid use and prostate cancer risk reduction and incidence are warranted based on the findings from this study. Controls should be introduced to eliminate other weaknesses identified in this trial: (1) there was a lack of information about omega-3 fatty acid intake and whether or not the source was fish consumption or supplement; (2) other risk factors for prostate cancer (family history, inflammatory conditions, smoking, obesity) were not accounted for; and (3) results were based on a single blood sample.

## CONCLUSION

The warning from the Brasky study that recommendations to increase long-chain omega fatty acid intake should consider its potential risk is not to be ignored, and this opens the door for conversations among male patients with or at risk for prostate cancer and APs. However, a recommendation for or against the use of fish oil supplements or the incorporation of fatty fish into the diet must incorporate the body of evidence as a whole. It is also important to consider the other benefits of omega-3 intake such as potential cardiovascular benefits, improved eye health, and slower rates of cognitive decline in the elderly when advising for or against its use.

A clear algorithm for translating research on omega-3 fatty acid consumption in men at risk for or with prostate cancer into practice does not currently exist. However, the conflicting data provide an opportunity for strongly designed, innovative research that will definitely answer the question and provide a specific clinical direction about the use of omega-3 fatty acids in patients at risk for or with prostate cancer.

Quality research will strengthen the CAM body of evidence. Participation in research will provide APs with a unique opportunity to evolve the existing body of evidence and impact future patient outcomes. Presently, it seems prudent to consider advocating for or against the use of fatty fish intake or fish oil supplements on a case-by-case basis after consideration of all risks and benefits.
